# Childhood BMI in relation to microbiota in infancy and lifetime antibiotic use

**DOI:** 10.1186/s40168-017-0245-y

**Published:** 2017-03-03

**Authors:** K. Korpela, M. A. C. Zijlmans, M. Kuitunen, K. Kukkonen, E. Savilahti, A. Salonen, C. de Weerth, W. M. de Vos

**Affiliations:** 1 Immunobiology Research Program, Department of Bacteriology and Immunology, University of Helsinki, Haartmaninkatu 3, PO box 21, 00014 Helsinki, Finland; 2 Department of Developmental Psychology, Behavioural Science Institute, Radboud University Nijmegen, Nijmegen, The Netherlands; 3 Children’s Hospital, University of Helsinki and Helsinki University Central Hospital, Helsinki, Finland; 4 Skin and Allergy Hospital, Department of Paediatrics, Helsinki University Central Hospital, Helsinki, Finland; 5 Laboratory of Microbiology, Wageningen University, Wageningen, The Netherlands

**Keywords:** Early-life microbiota, Childhood overweight, Bifidobacteria, Metabolic programming, Microarray

## Abstract

**Background:**

Children with high body mass index (BMI) at preschool age are at risk of developing obesity. Early identification of factors that increase the risk of excessive weight gain could help direct preventive actions. The intestinal microbiota and antibiotic use have been identified as potential modulators of early metabolic programming and weight development. To test if the early microbiota composition is associated with later BMI, and if antibiotic use modifies this association, we analysed the faecal microbiota composition at 3 months and the BMI at 5–6 years in two cohorts of healthy children born vaginally at term in the Netherlands (*N* = 87) and Finland (*N* = 75). We obtained lifetime antibiotic use records and measured weight and height of all children.

**Results:**

The relative abundance of streptococci was positively and the relative abundance of bifidobacteria negatively associated with the BMI outcome. The association was especially strong among children with a history of antibiotic use. *Bacteroides* relative abundance was associated with BMI only in the children with minimal lifetime antibiotic exposure.

**Conclusions:**

The intestinal microbiota of infants are predictive of later BMI and may serve as an early indicator of obesity risk. Bifidobacteria and streptococci, which are indicators of microbiota maturation in infants, are likely candidates for metabolic programming of infants, and their influence on BMI appears to depend on later antibiotic use.

**Electronic supplementary material:**

The online version of this article (doi:10.1186/s40168-017-0245-y) contains supplementary material, which is available to authorized users.

## Background

Overweight in childhood is an increasing global health problem with complex aetiology [1] and long-term consequences on the individual’s health, as it predisposes to cardiovascular risk factors, such as diabetes, hyperlipidemia, and increased blood pressure [1], and represents an important risk factor for adulthood obesity [2]. Pre- and perinatal maternal and environmental factors are being recognized as important contributors to the long-term metabolic programming and weight development of infants, and multiple lines of evidence indicate that childhood overweight may be strongly dependent on early-life influences [3]. The intestinal microbiota, acquired initially during birth from the mother and nurtured by breast milk, are emerging as an important modulator of early metabolic programming.

Mouse studies indicate a causal role for the intestinal microbiota regulating the development of growth, energy metabolism, fat accumulation, and susceptibility to diet-induced adiposity [4–6], a phenomenon termed microbiota-induced obesity [7]. Intestinal microbiota composition in infancy [8–12] and antibiotic use in early childhood [13–17] have been repeatedly associated with subsequent weight development in children. Mouse studies have established that early-life microbiota disruption by antibiotics causes increased growth and adiposity [6, 18, 19]. In addition to antibiotics, other microbiota-disrupting factors, such as birth by Caesarean section and short duration of breastfeeding, convey an increased risk of overweight, which is likely mediated by the early intestinal microbiota development [20, 21].

In mice, antibiotic-induced weight gain involves similar hormonal changes as diet-induced weight gain: increased levels of leptin and decreased levels of ghrelin and peptide YY [6]. The common factor between an obesogenic diet and antibiotic use is that they both strongly alter the intestinal microbiota, which have the capacity to regulate the host’s energy homeostasis via, e.g., FXR and TGR5 signalling by bile acids [22, 23], TLR4 signalling by lipopolysaccharide (LPS) [24, 25], Angptl4/Fiaf signalling [4, 26], and gut hormone and adipose tissue regulation by short-chain fatty acid (SCFA) production [27]. It is thus becoming increasingly evident that the early-life microbiota are involved in long-lasting metabolic programming.

We have previously discovered that the early-life use of macrolide antibiotics, which strongly modifies the microbiota, is a prerequisite for later antibiotic-associated increase in BMI in healthy Finnish children [28]. We have also reported recently that long duration of breastfeeding is negatively associated with BMI at preschool age but only among children who did not receive antibiotics before weaning, i.e., whose microbiota were intact [29], suggesting that the microbiota mediate the beneficial metabolic effects of breastfeeding. We therefore hypothesised that early microbiota composition may be associated with BMI in later childhood and that antibiotic use may modify this effect. In children with low lifetime antibiotic use, the early microbiota composition may be associated with the child’s susceptibility to diet-induced overweight, potentially involving leptin sensitivity or adipocyte programming [3]. In children with frequent lifetime antibiotic use, BMI is likely to be influenced not only by diet but also by the antibiotics; the early-life microbiome may thus affect the child’s susceptibility to both diet-induced and antibiotic-associated weight gain.

To test these hypotheses, we monitored a total of 162 children in the Netherlands and Finland, from birth to 5–6 years of age and analysed their total faecal microbiota at the age of 3 months using a culture-independent phylogenetic microarray. A consistent set of intestinal bacteria at 3 months of age was found to predict the BMI at 5–6 years, depending on the lifetime antibiotic use. While country-specific microbiota composition was noticeable, the BMI-associated signatures were evident independent of the geographic location.

## Methods

### Participants

The data consist of Dutch (*N* = 87) and Finnish (*N* = 75) children comprising 162 children in total (Table 1). These were selected from large longitudinal cohorts, the Bibo cohort (*N* = 193) in the Netherlands and the Flora cohort (*N* = 1223) in Finland, which have been reported previously [30, 31]. The selection criteria of the original studies included birth at term and no serious pregnancy or birth complications. For the current study, we selected vaginally born cases with appropriately stored faecal sample collected at 3 months and information on lifetime antibiotic use and BMI at 5–6 years. The children were measured for weight and height at the age of 5 (Flora) or 6 (Bibo) years.

**Table 1:** Characteristics of the cohorts (mean ± standard deviation)

	Finnish	Dutch
*N*	75	87
Birth weight (kg)	3.50 ± 0.44	3.64 ± 0.46
Growth first 6 months (kg); *N*	4.36 ± 075; 55	4.14 ± 0.69; 56
Duration of breastfeeding (weeks)	31.24 ± 17.19	14.18 ± 11.10
Total number of antibiotic courses	5.52 ± 4.82	2.67 ± 2.93
BMI	15.72 ± 1.54	15.75 ± 1.47

In the Flora study, pregnant mothers, whose infants had increased risk for allergy (at least one parent had a diagnosed allergic disease), were recruited at antenatal clinics and through advertisements in the Helsinki (Finland) suburban area. The Flora study was a probiotic intervention study, and here, we only included infants from the control group. The study was approved by the ethical committee of the Helsinki region hospital district. The Bibo study is a longitudinal study in which mothers and their children were followed from the third trimester of pregnancy on. Pregnant women were recruited through midwife practices in Nijmegen and surrounding areas (the Netherlands).

The median birth weight was 3.4 kg (range 2.3–4.7 kg) among the Finnish infants and 3.6 kg (range 2.7–4.7 kg) among the Dutch infants. In the Finnish cohort, 39 infants were first-born, and in the Dutch cohort, 23 infants were first-born. At the age of 3 months, 12 Finnish infants and 42 Dutch infants were no longer breastfed. The sub-cohorts analysed here did not differ from the total Flora and Bibo cohorts in terms of birth weight, BMI, or duration of breastfeeding. In the total Flora cohort, the mean ± sd birth weight was 3.59 ± 0.49 kg, duration of breastfeeding was 8.42 ± 5.23 months, and BMI at 5 years was 15.86 ± 1.38. In the total Bibo cohort, the mean ± sd birth weight was 3.61 ± 0.47 kg, duration of breastfeeding was 4.19 ± 2.78 months, and BMI at 6 years was 15.64 ± 1.35.

### Procedure

**Fig. 1:**
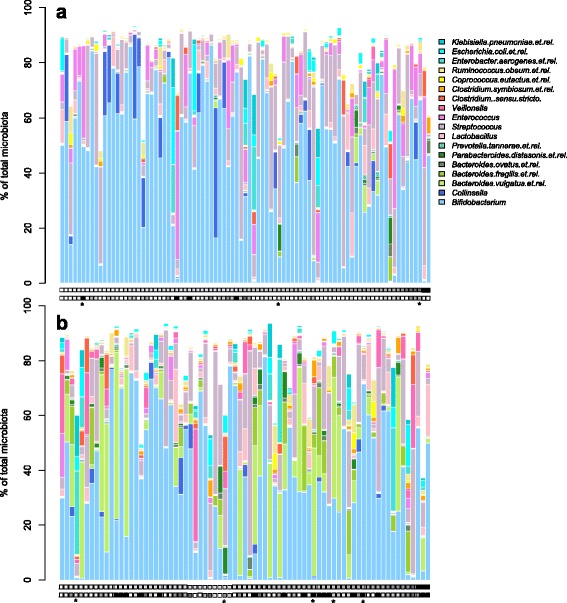
Microbiota composition of 3-month-old infants in the Dutch (**a**) and Finnish (**b**) cohorts. The 16 most abundant genus-level taxa are shown. Colour codes are from top down on each column. Each column represents an individual child, and the *squares* below the columns show the BMI of the child at 5–6 years (*white* = 13, *black* = 21) and lifetime antibiotic use (*white* = 0 courses, *black* = 8 or more courses). *Asterisks* indicate infants who had received a course of antibiotics before sample collection

A faecal sample was collected from all infants at the age of 3 months, by parents at home, as previously detailed [30, 31]. In addition, information on weight development from birth to 6 months of age was available for a subset of infants in both cohorts (Table 1). Growth during the first 6 months was calculated as change in weight from birth to age 6 months. BMI at 5–6 years was calculated based on the weight and height measured by a paediatrician or a researcher. Lifetime antibiotic use information was available for both cohorts. For the Dutch cohort, data on antibiotic prescriptions were obtained from clinical records from the child’s GP. For the Finnish children, the parents provided information on the number of antibiotic courses the child had received every 3–6 months during the first year, thereafter yearly in questionnaires. The overwhelming majority of the antibiotic courses were given to the children after the collection of the faecal samples; only eight infants received antibiotics before the age of 3 months (Fig. 1). The type of antibiotics used was not available for all infants, and therefore, only the total number of courses was used in the analysis. The level of antibiotic use was higher in the Finnish cohort: the median number of courses was 5 in the Finnish cohort and 2 in the Dutch cohort, corresponding to population-level data from both countries [32].

### Microbiota analysis

The faecal samples of both cohorts were processed and analysed using a pipeline that has been used in many infant and child microbiota studies [31, 33]. This included DNA extraction using the repeated bead beating method [34] and analysis of the microbiota composition using the HITChip phylogenetic microarray, which contains oligonucleotide probes for hypervariables regions on the 16S rRNA gene [35]. All samples were analysed on two independent microarray experiments, and the data only passed the quality control if the inter-experiment Pearson correlation was >0.97. The signal intensities were normalized using the fRPA method [36] and summarised at different levels of phylogenetic resolution: species, genus, and phylum, except for the *Firmicutes*, which was further divided to *Clostridium* clusters and *Bacilli*. Relative normalized signal intensities were calculated for all samples and all three levels. For the analyses, only bacterial groups with >30% prevalence at a minimum of 0.01% relative abundance were included (Additional file 1).

### Statistical analysis

Differences between the countries in bacterial relative abundances were tested using generalised linear models with negative binomial distribution. Associations between bacterial taxa to BMI and early growth were tested using correlations. To adjust for the effects of country, birth weight, breastfeeding duration, and antibiotic use on BMI, we calculated the deviance from the expected BMI, based on a linear model with the aforementioned variables. We then tested for a correlation between the BMI deviance and the log-transformed relative abundances of the bacterial taxa. The models were run using the whole data set and separately for each cohort to ascertain consistency in the associations. In addition, the models were run separately for children with minimal antibiotic exposure (0–1 lifetime courses), and those with several antibiotic courses, to see if the associations were present regardless of antibiotic exposure. Only associations that were significant in the total cohort (*p* < 0.05) and nearly significant (*p* < 0.15) in both countries separately were considered robust. All statistical analyses were conducted in R [37] using the package vegan [38]. R script for the analysis is available in Additional file 2.

## Results

### Country differences

A total of 162 intestinal microbiota profiles from 3-month-old infants in the Netherlands and Finland were analysed in duplicate using an established phylogenetic microarray (Fig. 1). In the analysis, a total of 115 bacterial taxa (58 genus-like groups, 14 *Clostridium* cluster/phylum groups, and 43 species-like groups) passed the abundance and prevalence filter and were included.

Although the faecal samples were processed and analysed in the same pipeline and platform, the microbiota compositions differed clearly between the countries (Fig. 1, see Additional file 3). Out of the 115 taxa, the relative abundance of 87 (76%) differed significantly between the countries. Country explained 13% of the inter-individual differences in microbiota composition (*p* = 0.001). The most pronounced difference was the relative abundance of the genus *Bacteroides*, which was at a low level in the Dutch infants, but very abundant in some, but not all, Finnish infants (Fig. 1, *p* < 0.00001). *Veillonella* showed a similar pattern (Fig. 1, *p* < 0.00001). Bacteria related to *Enterococcus* formed a major component of the microbiota in many Dutch infants, but represented a minority in the Finnish infants (Fig. 1, *p* < 0.00001). *Bifidobacterium* and *Streptococcus* were the dominant taxa in the intestinal microbiota of both cohorts with no significant cohort difference in relative abundance (*p* = 0.09 and *p* = 0.44, respectively).

### Association between early-life microbiota composition and BMI at the age of 5–6 years

BMI distributions did not markedly differ between the cohorts (see Additional file 4), and most children were normal weight (BMI 14–17, according to the CDC growth charts). In both cohorts, there were also overweight children (14 Dutch and 14 Finnish children with BMI >17) and underweight children (10 Dutch and 7 Finnish children with BMI <14).

The background variables country, birth weight, breastfeeding duration, and total lifetime antibiotic use explained 11% of the BMI variation. The only significant contributor to BMI was birth weight (*p* = 0.0005). Antibiotic use had a nearly significant positive association with BMI (*p* = 0.06).

**Fig. 2:**
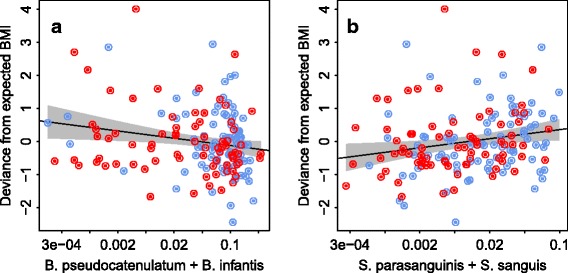
Association between selected bifidobacteria (**a**) and streptococci (**b**) at 3 months of age and deviance from expected BMI at 5–6 years in Finnish (*red*) and Dutch (*blue*) children. The deviance from expected is calculated based on birth weight and breastfeeding duration. See Table 2 for details. The trend lines (*shading*) show linear regression (95% confidence interval)

**Fig. 3:**
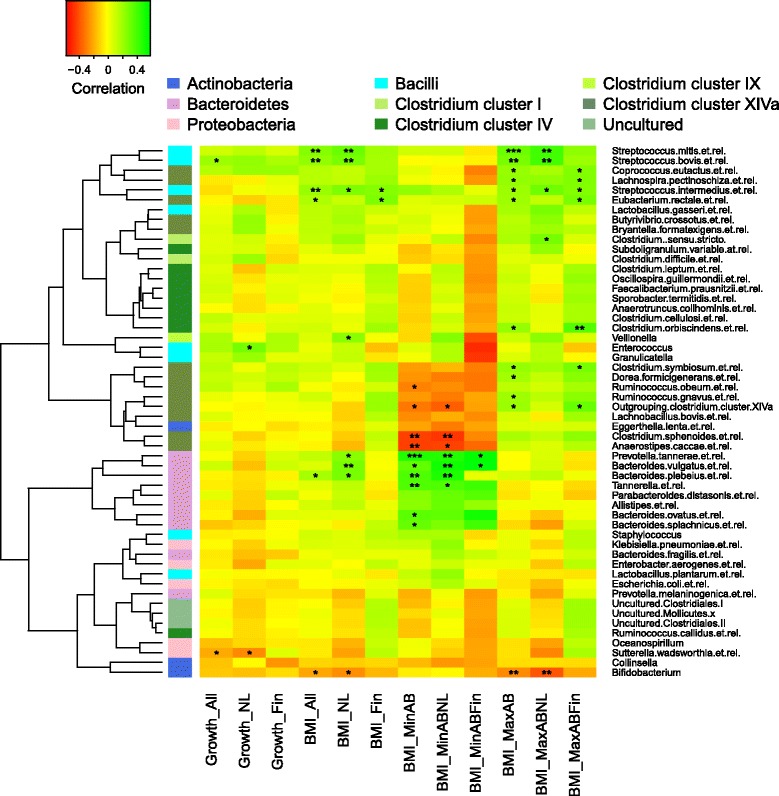
Association between genus-like bacterial groups with infant growth from birth to 6 months and BMI at 5–6 years in the total cohort, separately in the Dutch (NL) and Finnish (Fin) children and separately in children with minimal lifetime antibiotic use (minAB, 0–1 courses) and those with several antibiotic courses (maxAB, >1 courses)

The relative abundance of the phylum *Actinobacteria* was negatively and the relative abundance of the phylum *Firmicutes* was positively associated with BMI (Table 2). Among *Actinobacteria*, the most abundant genus *Bifidobacterium*—particularly the species *B. infantis*, *B. pseudocatenulatum*, *B. longum*, and *B. thermophilum*—was negatively associated with BMI (Table 2, Fig. 2a). Among *Firmicutes*, the class *Bacilli*, and particularly streptococci, were positively associated with BMI (Table 2, Fig. 2b). However, these associations varied by antibiotic exposure and were not present in the children with minimal antibiotic exposure (Table 2, Fig. 3). Among the children with minimal antibiotic exposure, there was a strong positive association between BMI and *Bacteroidetes*, particularly species related to *Bacteroides ovatus*, *Bacteroides vulgatus*, and *Prevotella tannerae* (Table 2, Fig. 3). Conversely, the phylum *Firmicutes* showed a significant positive association with BMI only among the children with a history of several antibiotic courses (Table 2, Fig. 3).

**Table 2:** Correlations (95% confidence interval) between bacterial taxa and BMI, adjusted for birth weight and duration of breastfeeding, in the total cohort (*N* = 162), in children with minimal antibiotic exposure (0–1 lifetime courses, *N* = 50), and children with several antibiotic courses (*N* = 112).

Taxon	All	*p*	Minimal AB	*p*	Several AB	*p*
*Actinobacteria*	−0.21 (−0.36 to −0.06)	0.01	−0.05 (−0.33 to 0.25)	0.76	−0.28 (−0.45 to −0.09)	<0.01
*Bifidobacterium*	−0.21 (−0.36 to −0.05)	0.01	−0.05 (−0.33 to 0.25)	0.76	−0.27 (−0.44 to −0.09)	<0.01
*Bifidobacterium infantis*	−0.19 (−0.34 to −0.04)	0.02	−0.05 (−0.33 to 0.25)	0.76	−0.23 (−0.4 to −0.04)	0.02
*Bifidobacterium longum*	−0.2 (−0.34 to −0.04)	0.02	−0.04 (−0.33 to 0.25)	0.77	−0.24 (−0.41 to −0.05)	0.01
*Bifidobacterium pseudocatenulatum*	−0.16 (−0.31 to 0)	0.05	0.04 (−0.25 to 0.33)	0.78	−0.25 (−0.42 to −0.06)	0.01
*Bifidobacterium thermophilum*	−0.16 (−0.31 to 0)	0.05	0.06 (−0.23 to 0.34)	0.69	−0.24 (−0.41 to −0.05)	0.01
*Bacteroidetes*	0.05 (−0.11 to 0.2)	0.57	0.3 (0.01 to 0.55)	0.04	−0.02 (−0.21 to 0.18)	0.88
*Bacteroides*	0.06 (−0.1 to 0.21)	0.5	0.31 (0.02 to 0.55)	0.04	0 (−0.19 to 0.19)	0.99
*Bacteroides ovatus et rel*	0.05 (−0.11 to 0.2)	0.57	0.36 (0.07 to 0.59)	0.02	−0.04 (−0.23 to 0.15)	0.69
*Bacteroides vulgatus et rel*	0.07 (−0.09 to 0.22)	0.41	0.34 (0.06 to 0.57)	0.02	−0.01 (−0.2 to 0.18)	0.92
*Prevotella tannerae et rel*	0.13 (−0.03 to 0.28)	0.11	0.49 (0.23 to 0.68)	0	−0.01 (−0.2 to 0.18)	0.91
*Firmicutes*	0.23 (0.08 to 0.38)	<0.01	−0.05 (−0.33 to 0.25)	0.76	0.34 (0.16 to 0.5)	<0.01
Clostridia	0.08 (−0.09 to 0.23)	0.36	−0.26 (−0.51 to 0.03)	0.08	0.21 (0.02 to 0.39)	0.03
*Lachnospira pectinoschiza et rel*	0.15 (−0.01 to 0.31)	0.06	0.02 (−0.27 to 0.31)	0.9	0.23 (0.04 to 0.4)	0.02
*Bacilli*	0.25 (0.09 to 0.39)	<0.01	0.21 (−0.09 to 0.47)	0.17	0.25 (0.06 to 0.42)	0.01
*Streptococcus*	0.26 (0.11 to 0.4)	<0.01	0.07 (−0.22 to 0.35)	0.64	0.31 (0.13 to 0.48)	<0.01
*Streptococcus bovis et rel*	0.24 (0.08 to 0.38)	<0.01	−0.02 (−0.31 to 0.28)	0.92	0.29 (0.1 to 0.46)	<0.01
*Streptococcus intermedius et rel*	0.22 (0.06 to 0.36)	0.01	0.23 (−0.06 to 0.49)	0.12	0.21 (0.02 to 0.38)	0.03
*Streptococcus mitis*	0.22 (0.06 to 0.37)	0.01	0.24 (−0.05 to 0.5)	0.1	0.21 (0.02 to 0.38)	0.03
*Streptococcus mitis et rel*	0.26 (0.11 to 0.41)	<0.01	0.1 (−0.2 to 0.38)	0.52	0.32 (0.13 to 0.48)	<0.01
*Streptococcus mutans*	0.18 (0.02 to 0.33)	0.03	0.18 (−0.11 to 0.45)	0.22	0.18 (−0.01 to 0.36)	0.06
*Streptococcus parasanguinis*	0.21 (0.05 to 0.36)	0.01	0.21 (−0.09 to 0.47)	0.17	0.21 (0.02 to 0.39)	0.03
*Streptococcus pneumoniae*	0.19 (0.03 to 0.34)	0.02	0.07 (−0.22 to 0.36)	0.63	0.22 (0.03 to 0.39)	0.02
*Streptococcus salivarius*	0.24 (0.08 to 0.39)	<0.01	0.07 (−0.23 to 0.35)	0.65	0.28 (0.1 to 0.45)	<0.01
*Streptococcus sanguis*	0.28 (0.12 to 0.42)	<0.01	0.03 (−0.26 to 0.32)	0.82	0.34 (0.16 to 0.5)	<0.01

### Microbiota composition and early growth

The associations between BMI and microbiota composition were largely reflected in the associations between microbiota and early growth (Fig. 3). Early growth was positively associated with the relative abundance of *Firmicutes* at 3 months (correlation = 0.21 [0.03–0.39], *p* = 0.02), particularly *Clostridia* (0.20 [0.01–0.37], *p* = 0.04), and species related to *Streptococcus bovis* (0.2 [0.02–0.39], *p* = 0.03).

## Discussion

We compared the early-life faecal microbiota compositions in two cohorts of healthy infants and identified bacterial taxa whose relative abundances were consistently associated with later BMI in both the Dutch and the Finnish cohort.

Children with a low relative abundance of *Actinobacteria* and a high relative abundance of *Firmicutes* at the age of 3 months were likely to attain a high BMI at the age of 5–6 years, but only if they received several courses of antibiotics. This is in line with our earlier finding that macrolide use in early life, which we have shown to nearly eliminate *Actinobacteria* in children, is required for the later antibiotic-associated increase in BMI to occur [28]. These results suggest that the early-life microbiota composition may modify the later metabolic response to antibiotics. Antibiotic use is associated with an increase in LPS-producing Gram-negative bacteria [28], and frequent antibiotic use may thus involve recurrent LPS surges, similar to a high-fat diet [39]. It is possible that the host’s metabolic responses to this depend on early-life microbial exposure.

Two Finnish studies based on another birth cohort have noted a negative association between the relative abundance of *Bifidobacterium* in infancy and later BMI [8, 9]. Moreover, high relative abundance of *Firmicutes* and low relative abundance of bifidobacteria has been associated with rapid growth, an obesity predictor [40], and with increased adiposity in infancy [11, 41]. Here, we found the same associations particularly among the children with a history of antibiotic use. Streptococci, which were positively associated with early growth and later BMI, form a significant component of the neonate gut microbiota [31] and inhabit the small intestine in adults [42]. Their relative abundance in faecal microbiota normally declines rapidly during the first weeks, as they are replaced by anaerobic bifidobacteria [31]. High relative abundance of streptococci and low relative abundance of bifidobacteria at 3 months likely reflects an altered maturation of the microbiota. It is possible that the microbiota composition in infancy is a reflection of, rather than a causal contributor to, the physiological, metabolic, or immunological development of the host. There are, however, indications that the microbiota actively participate in early-life metabolic programming [4–6].

Increasing amount of evidence is indicating that bifidobacteria have an important role in the host’s energy metabolism. Rodent studies have shown that the negative metabolic effects of a high-fat diet are dependent on a reduction in the abundance of bifidobacteria [43–45]. Indeed, a bifidogenic synbiotic has been shown to reduce weight in overweight children [46]. Obesity and high-fat diets induce endotoxemia, an increase in the level of circulating lipopolysaccharide (LPS), which appears to be a requirement for the associated metabolic consequences [39, 43, 46]. Bifidobacteria may reduce systemic inflammation by reducing the abundance of inflammatory LPS-producing bacteria [45], by up-regulating tight-junction proteins in the gut and thus reducing the leakage of LPS and other bacterial antigens into the circulation [43, 47], and by stimulating regulatory T cells and the production of anti-inflammatory cytokines [45]. In obese adults, *Bifidobacterium* spp. correlate negatively with serum LPS, cholesterol, and fat mass [48].

*B. pseudocatenulatum* and *B. longum* have previously been shown to reduce weight gain and the metabolic effects of a high-fat diet in rodents, causing lower cholesterol and leptin levels and improved insulin sensitivity [45, 49–51]. The mechanism is at least partly related to the amelioration of the diet-induced endotoxemia and inflammation in the gut, liver, and in adipose tissue [45, 50, 51], and the deconjugation of bile acids [52], which influences the host’s lipid metabolism and energy expenditure [23]. *B. pseudocatenulatum* has been shown to cause changes in the expression of hepatic genes involved in lipid metabolism [53].

In previous Dutch studies, *Bacteroides* species, and particularly *B. fragilis* have been associated with higher BMI outcomes in later childhood [10, 12]. We found the same association, but only in the children with minimal antibiotic exposure. Dutch children generally use antibiotics less frequently than Finnish children, which may explain why this association has previously not been observed in Finnish children. *Bacteroides* spp. were generally more abundant in the Finnish infants.

## Conclusions

While our results do not establish causation between early-life microbiota composition and later BMI, they show that the microbiota may represent a biomarker for assessing individual risks of excessive weight gain. The results support the emerging paradigm of microbiota-dependent metabolic programming in humans.

## Additional files

Additional file 1: Relative abundances of bacterial groups in each sample. (XLSX 316 kb)
Additional file 2: R script for the main analysis. (R 4 kb)
Additional file 3: Bacterial taxa with significantly different relative abundances in the Dutch and Finnish cohorts. All FDR-corrected *p* values are <0.05. (DOCX 145 kb)
Additional file 4: BMI distribution in the Finnish and Dutch cohorts. (PDF 117 kb)
