# Supplementary material for: Childhood BMI in relation to microbiota in infancy and lifetime antibiotic use
Source: Microbiome. 2017 Mar 3;5:26. doi: 10.1186/s40168-017-0245-y (PMC5335838; doi:10.1186/s40168-017-0245-y)
Supplement: Additional file 3: — Bacterial taxa with significantly different relative abundances in the Dutch and Finnish cohorts. All FDR-corrected p values are <0.05. (DOCX 145 kb) [file 40168_2017_245_MOESM3_ESM.docx]

| Phylum | Intermediate taxonomic level | Taxon | Fold Change (NL/Fin) | p |
| --- | --- | --- | --- | --- |
| Actinobacteria | Actinobacteria | Actinobacteria | 1.426 | 0.035 |
| Actinobacteria | Actinobacteria | Bifidobacterium adolescentis | 13.168 | <0.001 |
| Actinobacteria | Actinobacteria | Bifidobacterium animalis | 126.980 | <0.001 |
| Actinobacteria | Actinobacteria | Bifidobacterium bifidum | 26.296 | <0.001 |
| Actinobacteria | Actinobacteria | Bifidobacterium catenulatum | 18.003 | <0.001 |
| Actinobacteria | Actinobacteria | Bifidobacterium dentium | 44.803 | <0.001 |
| Actinobacteria | Actinobacteria | Bifidobacterium gallicum | 74.960 | <0.001 |
| Actinobacteria | Actinobacteria | Bifidobacterium infantis | 1.775 | 0.005 |
| Actinobacteria | Actinobacteria | Bifidobacterium longum | 1.675 | 0.011 |
| Actinobacteria | Actinobacteria | Bifidobacterium pseudolongum | 0.581 | 0.003 |
| Actinobacteria | Actinobacteria | Bifidobacterium thermophilum | 6.766 | <0.001 |
| Actinobacteria | Actinobacteria | Collinsella | 4.110 | <0.001 |
| Actinobacteria | Actinobacteria | Collinsella aerofaciens | 6.852 | <0.001 |
| Actinobacteria | Actinobacteria | Eggerthella lenta et rel | 2.446 | 0.001 |
| Bacteroidetes | Bacteroidetes | Bacteroidetes | 0.269 | <0.001 |
| Bacteroidetes | Bacteroidetes | Bacteroides | 0.181 | <0.001 |
| Bacteroidetes | Bacteroidetes | Bacteroides fragilis et rel | 0.133 | <0.001 |
| Bacteroidetes | Bacteroidetes | Bacteroides plebeius et rel | 1.581 | <0.001 |
| Bacteroidetes | Bacteroidetes | Bacteroides splachnicus et rel | 2.763 | <0.001 |
| Bacteroidetes | Bacteroidetes | Bacteroides vulgatus et rel | 0.037 | <0.001 |
| Bacteroidetes | Bacteroidetes | Parabacteroides distasonis et rel | 0.517 | 0.007 |
| Bacteroidetes | Bacteroidetes | Prevotella melaninogenica et rel | 0.429 | <0.001 |
| Firmicutes | Bacilli | Bacilli | 1.361 | 0.023 |
| Firmicutes | Bacilli | Enterococcus | 4.927 | <0.001 |
| Firmicutes | Bacilli | Enterococcus faecalis | 15.205 | <0.001 |
| Firmicutes | Bacilli | Lactobacillus gasseri et rel | 4.654 | <0.001 |
| Firmicutes | Bacilli | Staphylococcus | 4.653 | <0.001 |
| Firmicutes | Bacilli | Streptococcus equinus | 3.325 | <0.001 |
| Firmicutes | Bacilli | Streptococcus intermedius et rel | 10.290 | <0.001 |
| Firmicutes | Bacilli | Streptococcus mitis | 4.668 | <0.001 |
| Firmicutes | Bacilli | Streptococcus mutans | 21.452 | <0.001 |
| Firmicutes | Bacilli | Streptococcus parasanguinis | 7.793 | <0.001 |
| Firmicutes | Bacilli | Streptococcus pneumoniae | 0.259 | <0.001 |
| Firmicutes | Bacilli | Streptococcus pyogenes | 23.559 | <0.001 |
| Firmicutes | Bacilli | Streptococcus salivarius | 2.095 | <0.001 |
| Firmicutes | Bacilli | Streptococcus thermophilus | 0.208 | <0.001 |
| Firmicutes | Bacilli | Streptococcus viridans | 5.115 | <0.001 |
| Firmicutes | Clostridia | Clostridia | 0.612 | <0.001 |
| Firmicutes | Clostridium cluster IV | Anaerotruncus colihominis et rel | 0.858 | <0.001 |
| Firmicutes | Clostridium cluster IV | Clostridium cellulosi et rel | 0.588 | <0.001 |
| Firmicutes | Clostridium cluster IV | Clostridium leptum et rel | 0.557 | <0.001 |
| Firmicutes | Clostridium cluster IV | Clostridium orbiscindens et rel | 0.599 | <0.001 |
| Firmicutes | Clostridium cluster IV | Faecalibacterium prausnitzii et rel | 0.640 | <0.001 |
| Firmicutes | Clostridium cluster IV | Oscillospira guillermondii et rel | 0.548 | <0.001 |
| Firmicutes | Clostridium cluster IV | Ruminococcus callidus et rel | 0.763 | <0.001 |
| Firmicutes | Clostridium cluster IV | Sporobacter termitidis et rel | 0.617 | <0.001 |
| Firmicutes | Clostridium cluster IV | Subdoligranulum variable at rel | 0.585 | <0.001 |
| Firmicutes | Clostridium cluster IX | Clostridium cluster IX | 0.138 | <0.001 |
| Firmicutes | Clostridium cluster IX | Veillonella | 0.053 | <0.001 |
| Firmicutes | Clostridium cluster XI | Anaerovorax odorimutans et rel | 0.813 | 0.001 |
| Firmicutes | Clostridium cluster XIVa | Anaerostipes caccae et rel | 0.444 | <0.001 |
| Firmicutes | Clostridium cluster XIVa | Bryantella formatexigens et rel | 2.206 | <0.001 |
| Firmicutes | Clostridium cluster XIVa | Butyrivibrio crossotus et rel | 2.325 | <0.001 |
| Firmicutes | Clostridium cluster XIVa | Clostridium cluster XIVa | 0.708 | 0.010 |
| Firmicutes | Clostridium cluster XIVa | Clostridium sphenoides et rel | 0.524 | <0.001 |
| Firmicutes | Clostridium cluster XIVa | Clostridium symbiosum et rel | 0.499 | <0.001 |
| Firmicutes | Clostridium cluster XIVa | Eubacterium rectale et rel | 0.591 | 0.001 |
| Firmicutes | Clostridium cluster XIVa | Lachnobacillus bovis et rel | 0.654 | <0.001 |
| Firmicutes | Clostridium cluster XIVa | Lachnospira pectinoschiza et rel | 1.801 | <0.001 |
| Firmicutes | Clostridium cluster XIVa | Ruminococcus gnavus et rel | 0.493 | <0.001 |
| Firmicutes | Clostridium cluster XIVa | Ruminococcus obeum et rel | 0.627 | <0.001 |
| Firmicutes | Clostridium cluster XVI | Clostridium cluster XVI | 0.949 | 0.006 |
| Firmicutes | Clostridium cluster XVIII | Clostridium cluster XVIII | 0.739 | 0.024 |
| Firmicutes | Uncultured Clostridiales I | Uncultured Clostridiales I | 0.742 | <0.001 |
| Firmicutes | Uncultured Clostridiales II | Uncultured Clostridiales II | 0.744 | <0.001 |
| Firmicutes | Uncultured Mollicutes | Uncultured Mollicutes | 0.860 | 0.002 |
| Proteobacteria | Proteobacteria | Oceanospirillum | 4.543 | <0.001 |
| Proteobacteria | Proteobacteria | Sutterella wadsworthia et rel | 3.395 | <0.001 |
