# Supplementary figures and images for: Childhood BMI in relation to microbiota in infancy and lifetime antibiotic use
Source: Microbiome. 2017 Mar 3;5:26. doi: 10.1186/s40168-017-0245-y (PMC5335838; doi:10.1186/s40168-017-0245-y)

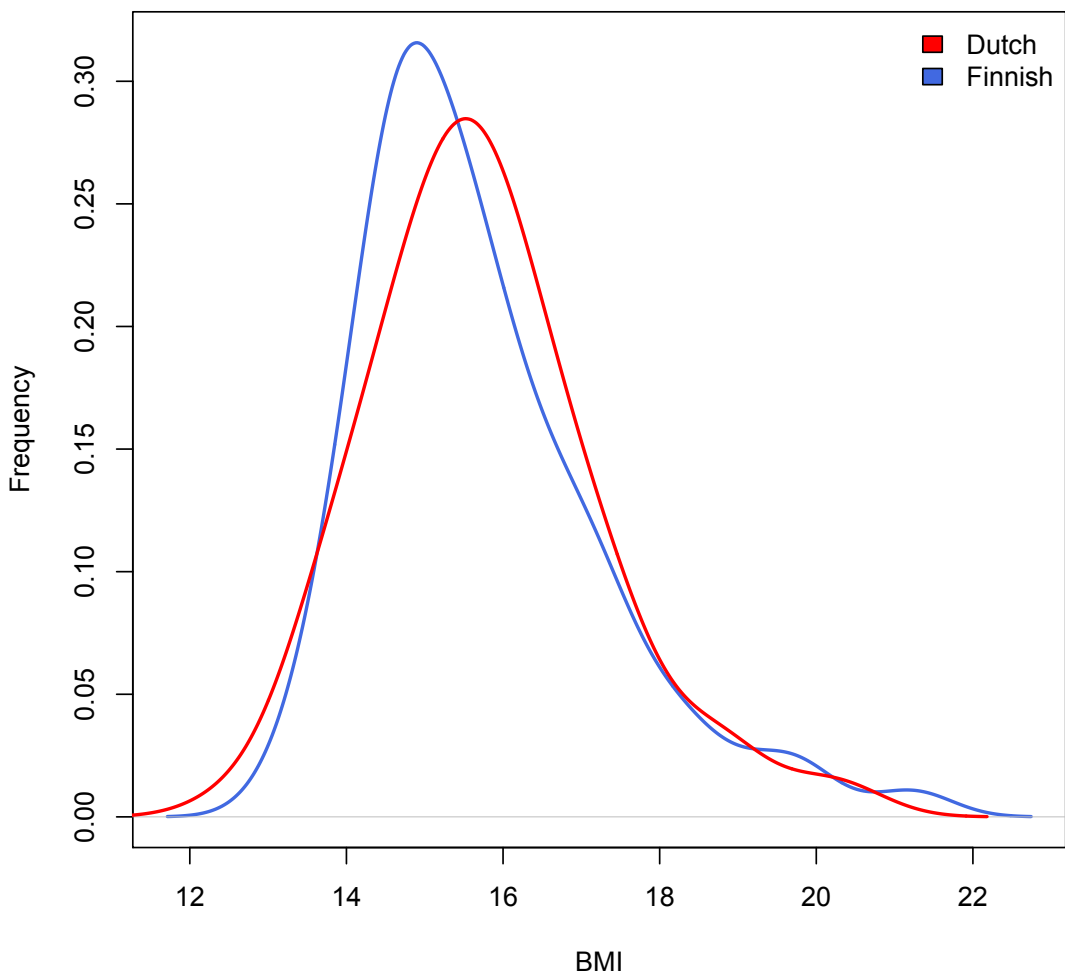

Supplement: Additional file 4: — BMI distribution in the Finnish and Dutch cohorts. (PDF 117 kb) [file 40168_2017_245_MOESM4_ESM.pdf]
